# Evolution of embryonic developmental period in the marine bird families Alcidae and Spheniscidae: roles for nutrition and predation?

**DOI:** 10.1186/1471-2148-10-179

**Published:** 2010-06-14

**Authors:** J Mark Hipfner, Kristen B Gorman, Rutger A Vos, Jeffrey B Joy

**Affiliations:** 1 Centre for Wildlife Ecology, Department of Biological Sciences, Simon Fraser University, 8888 University Drive, Burnaby, British Columbia, V5A 1S6, Canada; 2 Reading Evolutionary Biology Group, School of Biological Sciences, University of Reading, Philip Lyle Building Level 4, Reading, RG6 6BX, UK; 3 Department of Biological Sciences, Simon Fraser University, 8888 University Drive, Burnaby, British Columbia, V5A 1S6, Canada

## Abstract

**Background:**

Nutrition and predation have been considered two primary agents of selection important in the evolution of avian life history traits. The relative importance of these natural selective forces in the evolution of avian embryonic developmental period (EDP) remain poorly resolved, perhaps in part because research has tended to focus on a single, high taxonomic-level group of birds: Order Passeriformes. The marine bird families Alcidae (auks) and Spheniscidae (penguins) exhibit marked variation in EDP, as well as behavioural and ecological traits ultimately linked to EDP. Therefore, auks and penguins provide a unique opportunity to assess the natural selective basis of variation in a key life-history trait at a low taxonomic-level. We used phylogenetic comparative methods to investigate the relative importance of behavioural and ecological factors related to nutrition and predation in the evolution of avian EDP.

**Results:**

Three behavioural and ecological variables related to nutrition and predation risk (i.e., clutch size, activity pattern, and nesting habits) were significant predictors of residual variation in auk and penguin EDP based on models predicting EDP from egg mass. Species with larger clutch sizes, diurnal activity patterns, and open nests had significantly shorter EDPs. Further, EDP was found to be longer among birds which forage in distant offshore waters, relative to those that foraged in near shore waters, in line with our predictions, but not significantly so.

**Conclusion:**

Current debate has emphasized predation as the primary agent of selection driving avian life history diversification. Our results suggest that both nutrition and predation have been important selective forces in the evolution of auk and penguin EDP, and highlight the importance of considering these questions at lower taxonomic scales. We suggest that further comparative studies on lower taxonomic-level groups will continue to constructively inform the debate on evolutionary determinants of avian EDP, as well as other life history parameters.

## Background

Embryonic developmental period (EDP) is an evolutionarily conservative life-history trait in birds, with 80% of the Class-wide variation residing at the taxonomic levels of order and family-within-order [1,2]. Such strong conservatism is to be expected, given that embryonic development is a tightly constrained process [3], and that egg size, which has a positive relationship with EDP, is equally conservative [1,2]. Nonetheless, EDP varies more than threefold even among avian species with eggs of similar size [4], indicating that this trait can and does respond strongly to natural selection. However, accounting for this variation remains an ongoing challenge [5-8].

All else being equal, we expect natural selection to favor a reduction in the time taken to complete development [9]. However, there may be fitness costs for the individual associated with its faster development, such as decreases in resistance to pathogens and parasites [8,10,11] or breeding lifespan [12,13], which could counteract that tendency. How then are the evolutionary trade-offs resolved, and what ultimate factors play important roles? Nutrition and predation have been considered two primary agents of selection on avian life-history traits, including the rate of development [5,14]. However, their roles remain somewhat poorly resolved, perhaps in part because research has tended to focus on a single, high taxonomic-level group of birds: Order Passeriformes [7,15-20]. Further, several basic assumptions that underlie much of the passerine research, which in general supports a primary role for predation [14], have been questioned [15]. Here, we pose a simple question: could the focused study of lower taxonomic-level avian groups help clarify the relative roles of nutrition and predation in the evolution of EDP?

Lack [5] first noted that EDP and post-EDP are positively correlated across avian species. The underlying cause of the relationship remains obscure (e.g., pleiotropy or independent unidirectional selection), and one can find exceptions to the general rule [18,21]. Nonetheless, the relationship has been demonstrated in phylogenetically-controlled analyses [2], supporting its biological relevance. Lack proposed that the correlation indicated the existence of an evolutionary predisposition for a constant growth trajectory, an idea also supported by Bennett and Owens [2]. That a relatively constant rate of development [22] is ultimately beneficial, either fast or slow depending on critical facets of a species' ecology, is consistent with recent studies documenting deleterious, long-term phenotypic consequences associated with irregular growth [12]. Consequently, we can expect rapid embryonic development to evolve more readily in species where parents can provide sufficient nutrition to support rapid post-embryonic development. If so, then behavioural and ecological factors that increase the rate at which parents deliver food should lead to coevolution of a briefer EDP.

Clutch size is another factor that could affect EDP [23]. The intensity of sibling competition for food increases with brood size, and individuals that hatch early usually outcompete late-hatched siblings, especially if hatching is asynchronous. Therefore, we can expect from existing theory that a larger clutch size selects for a briefer EDP and brood reduction as nest-mates engage in an evolutionary race to hatch first [6,18].

Like nutrition, predation also has a potentially complex relationship with EDP. On the one hand, life-history theory suggests that long-lived species should accept an increased risk to their offspring when countered by a decrease in the risk to themselves. Thus, parents in long-lived species should be less attentive to the nest site if that attentiveness subjects them to the risk of being depredated. This could drive the species to slower overall development as a result of frequent egg neglect [7] (but see Tieleman et al. [19]). On the other hand, it is more commonly argued that an increase in the offspring's mortality rate while in the nest could select for more rapid development in order to minimize the period of vulnerability [5,17,24].

Among avian families, the Alcidae exhibit unusual variation in several behavioural and ecological traits ultimately linked to EDP in other avian groups [25]. Throughout all species within the family, both males and females incubate on approximately equal schedules and there is no feeding of incubating adults by non-incubating partners. Egg mass varies by a factor of four, while clutch size is either one or two, representing considerable variation in relative if not absolute terms. The family further exhibits unparalleled variation in the amount of time spent (species means of two-54 days), and post-EDP completed (zero to 80%), at the nest site before offspring depart to sea, either alone, or accompanied by one or both parents [1,26]. Variation in the frequency of offspring provisioning is also extreme: the auks include diurnal, nearshore-feeding species that bi-parentally deliver ten to 15 meals per day; but also nocturnal, offshore species that bi-parentally deliver at most two meals per day [27]. Several species even forego provisioning at the nest site altogether as their chicks are precocial [28] and fed at sea. Moreover, a range of nest types is used. Most auks breed on mammal-free islands in enclosed earthen burrows, or rock crevices, where their eggs are inaccessible to avian predators. However, some species breed in the open, either cryptically on old-growth tree branches or in alpine tundra, while others nest very densely and conspicuously on exposed cliff ledges. For open-nesting auks, the nest site is vulnerable to avian predators, and in some situations rates of predation on eggs and chicks can be so high as to compromise population viability [29,30].

The penguins, originally conflated with some auks for their morphological similarities (the now extinct great auk (*Pinguinus impennis*) was the original bird called penguin, from the Welsh *pen gwyin*, for white head [31]), and have comparable variation in the traits under study here. Like auks, in all penguin species, except the emperor penguin (*Aptenodytes forsteri*), both sexes take incubation shifts [32]. The lower extreme in egg mass is found in the little blue penguin (*Eudyptula minor*) (53 g), while the upper extreme is found in the emperor penguin (465 g) with most other species ranging between 100 and 150 g [32]. Clutch size is also one or two eggs [32]. Incubation period ranges between one and two months, while nestling period has some high extremes in the emperor and king (*Aptenodytes patagonicus*) penguin (150 days), with the rest of the species ranging between 49 and 90 days [32]. Like the auks, penguin nests vary from those built in crevices or burrows, to nests in the open built out of sticks and grass, to bare patches on the ground. Penguins also are vulnerable to avian predators, with low breeding success in small colonies being partially attributable to depredation [33].

**Figure 1:**
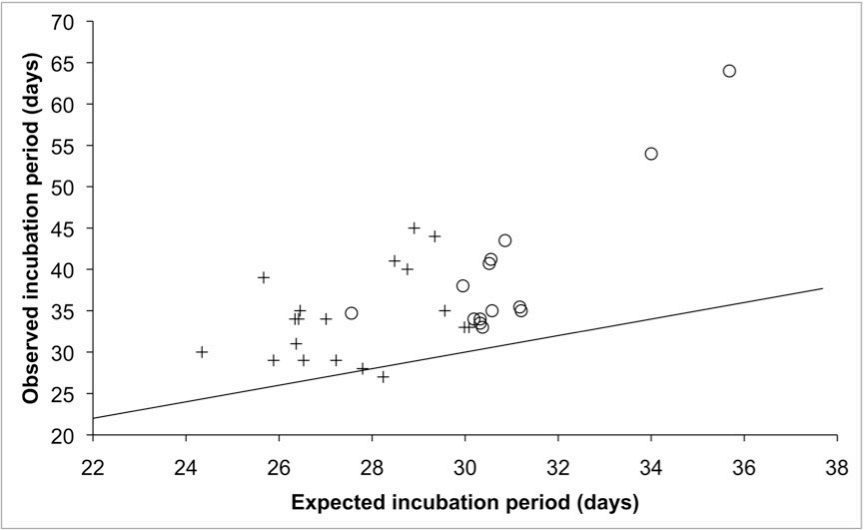
**Embryonic developmental periods (EDP) in the auks and penguins in relation to values predicted from fresh egg mass for Charadriiform birds**. The straight line represents a 1:1 relationship (i.e., observed EDP is the same as that predicted from egg mass). The equation for Charadriiform birds is: predicted EDP = 17.18 × egg mass^0.119 ^[74]. Alcids are indicated by +, penguins are indicated by °.

Given the extent of variation in that suite of ecological and life-history traits (Table 1), it is not surprising that EDP varies widely among auk and penguin species (27 to 64 days, or approximately zero-60% longer than predicted from egg mass; Figure 1). Therefore, these taxa provide a unique opportunity to assess variation in a key life-history trait at a low taxonomic-level. Here, we examine the influence of behavioural and ecological factors related to nutrition and predation risk in the evolution of EDPs within a comparative, phylogenetic framework. We test the following specific predictions: (1) species that provision nocturnally or crepuscularly, thus only once per day, will have longer EDPs than diurnal species; (2) species that feed in offshore habitats far from the centrally-located nest site will have longer EDPs than inshore species; (3) species that lay a single-egg clutch, thereby lacking sibling competition, will have longer EDPs than species with two-egg clutches; and (4) species that use enclosed nest sites where offspring are safer from predators will have longer EDPs than species that use open nest sites. We used a maximum likelihood analysis corrected for phylogeny [34] to study the regression of EDP residuals (over those predicted by egg mass) and our four behavioural and ecological predictor variables using phylogenetic hypotheses generated from our Bayesian phylogeny estimation.

**Table 1:** Taxon identifiers, behavioural, ecological, and life-history variables used in the analysis, in addition to GenBank accession numbers

Scientific name	Common name	**NCBI acc**^**1**^	**EDP**^**2**^	**EDPr**^**3**^	**EM**^**4**^	**CS**^**5**^	**FH**^**6**^	**AP**^**7**^	**NH**^**8**^
*Alca torda*	Razorbill	AJ242683	35	5.44	95.70	1	0	1	0
*Alle alle*	Dovekie	AJ242684	29	3.12	31.30	1	1	1	0
*Uria aalge*	Common murre	AJ242686	33	2.92	110.80	1	0	1	1
*U*. *lomvia*	Thick-billed murre	AJ242687	33	3.01	107.80	1	1	1	1
*Synthliboramphus wumizusume*	Japanese murrelet	U37306	31	4.63	36.60	2	1	0	0
*S*. *antiquus*	Ancient murrelet	U37303	34	6.99	44.80	2	1	0	0
*S*. *hypoleucus*	Xantus' murrelet	U37305	34	7.58	37.20	2	1	0	0
*Cepphus carbo*	Spectacled guillemot	U37292	27	-1.24	65.10	2	0	1	0
*C*. *Columba*	Pigeon guillemot	U37293	28	0.20	57.00	2	0	1	0
*C*. *grille*	Black guillemot	AJ242688	29	1.77	47.90	2	0	1	0
*Brachyramphus marmoratus*	Marbled murrelet	U63055	29	2.47	38.50	1	0	0	1
*Ptychoramphus aleuticus*	Cassin's auklet	U37302	39	13.33	29.20	1	1	0	0
*Aethia pusilla*	Least auklet	U37104	30	5.66	18.70	1	1	1	0
*A*. *cristatella*	Crested auklet	U37087	34	7.66	36.30	1	1	1	0
*Cyclorrhynchus psittacula*	Parakeet auklet	U37296	35	8.55	37.60	1	1	1	0
*Cerorhinca monocerata*	Rhinoceros auklet	U37295	45	16.10	79.20	1	1	0	0
*Fratercula corniculata*	Horned puffin	U37299	40	11.24	75.90	1	1	1	0
*F*. *arctica*	Atlantic puffin	U37297	41	12.52	70.00	1	1	1	0
*F*. *cirrhata*	Tufted puffin	U37298	44	14.65	90.00	1	1	1	0
*Aptenodytes forsteri*	Emperor penguin	DQ137225	64	28.32	465.00	1	1	1	1
*A*. *patagonicus*	King penguin	AY139623	54	20.00	310.00	1	1	1	1
*Eudyptula minor*	Little blue penguin	NC_004538	34.7	7.14	53.00	2	0	1	0
*Eudyptes chrysocome*	Rockhopper penguin	AF076051	34	3.68	118.40	2	1	1	1
*E*. *chrysolophus*	Macaroni penguin	AF076052	35.45	4.28	149.46	2	1	1	1
*E*. *pachyrhynchus*	Fiordland penguin	DQ137210	33.5	3.18	118.40	2	0	1	1
*E*. *sclateri*	Erect-crested penguin	DQ137209	35	3.80	150.70	2	-	1	1
*Pygoscelis adeliae*	Adelie penguin	GQ925801	33	2.63	120.10	2	0	1	1
*P*. *Antarctica*	Chinstrap penguin	AF076089	34	3.81	114.10	2	0	1	1
*P*. *papua*	Gentoo penguin	AF076090	35	4.42	127.08	2	0	1	1
*Spheniscus demersus*	Black-footed penguin	DQ137217	38	8.05	106.80	2	0	1	0
*S*. *humboldti*	Peruvian penguin	AY567916	40.7	10.18	125.00	2	0	1	0
*S*. *magellanicus*	Magellanic penguin	DQ137218	41.2	10.65	126.25	2	0	1	0
*Megadyptes antipodes*	Yellow-eyed penguin	DQ137224	43.5	12.64	137.20	2	-	1	1

^1^NCBI accession number; ^2^Embryonic development period; ^3^Embryonic development period residuals; ^4^Egg mass in grams; ^5^Clutch size; ^6^Foraging habitat (0 = inshore, 1 = offshore); ^7^Activity pattern (0 = nocturnal or crepuscular, 1 = diurnal); ^8^Nesting habits (0 = enclosed, 1 = open).

## Results

### Phylogenetic relationships

**Figure 2:**
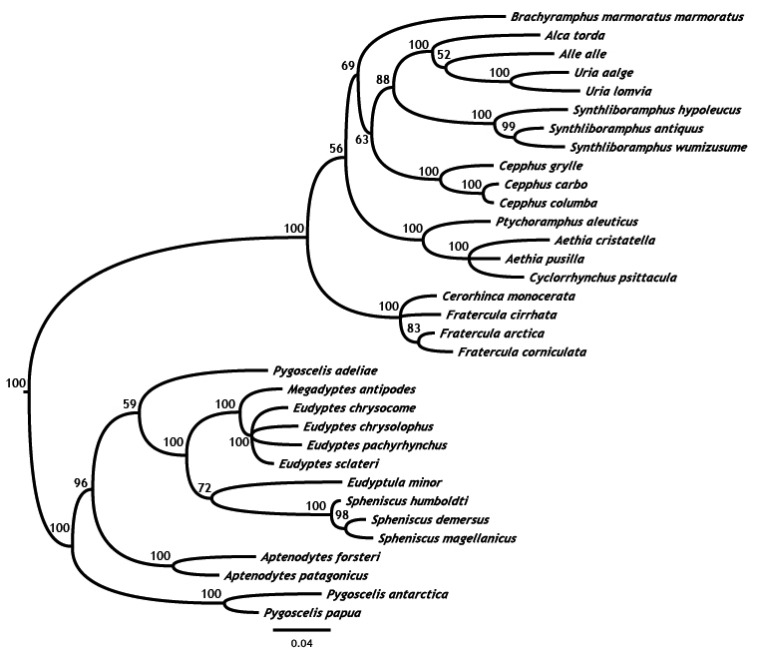
**Auk and penguin phylogenetic relationships**. Majority rule consensus tree, pruned to include species for which life history data were available, with Bayesian nodal posterior probabilities as inferred from mitochondrial cytochrome *b *gene sequence data.

During our MCMC phylogenetic inference we discarded those trees sampled during the first 2.5 million (out of ten million) generations, a very safe, long burn-in given the speed of convergence. The majority rule consensus over the full set of trees sampled after burn-in generally shows that most genera are reconstructed as monophyletic groups (the only exception in the consensus being the instability of the Adélie penguin (*Pygoscelis adeliae*), Figure 2) and that the topology matches the current understanding of the phylogeny of the taxa under study (e.g. see for comparison Pereira and Baker [35], Bertelli and Giannini [36], and Baker et al. [37]), albeit with some nodal instability for the deeper nodes, an effect caused by saturation of sites in the cytochrome *b *locus at this level of divergence.

### Comparative analysis

The estimate for λ = 0.999995 shows that the assumption of Brownian motion, which is made by many comparative analyses, holds. With an r^2 ^= 0.444949, our independent variables explained a large proportion of the variation in the dependent variable. Of our independent variables, clutch size had the greatest (negative) influence on EDP, a result that was highly significant (p < 0.01), confirming the prediction that an arms race between siblings for early hatching should drive the evolution of shorter EDP in species with two-egg clutches relative to those with one-egg clutches [6,18]. Activity pattern and nesting habits also both significantly affected EDP, in the direction predicted: diurnal taxa had shorter EDP, presumably due to their higher provisioning rates, and taxa with open nests had shorter EDP, presumably due to higher predation risk. Although the standardized coefficient for foraging habitat suggested an effect in the predicted direction (offshore foragers seem to have longer EDP), this effect was not significant (see Table 2 for analysis results).

**Table 2:** Regression analysis results

Variable	Standardized coefficient	Standard error
Clutch size	-8.337654**	2.552405
Offshore foraging	1.990214	1.575975
Diurnality	-3.497337*	1.695883
Open nests	-4.164798*	2.258906

Summary of the (phylogenetically corrected, reasonably assuming Brownian motion given λ = 0.999995) analysis of the regression of auk and penguin embryonic developmental period (EDP) residuals (computed from the EDP predicted from egg mass) on clutch size, foraging habitat (0 = inshore, 1 = offshore), activity pattern (0 = nocturnal or crepuscular, 1 = diurnal) and nesting habits (0 = enclosed, 1 = open). The independent variables explain a large amount of the variation in EDP (r^2 ^= 0.444949). Alpha = 28.483666 (s.e. = 5.898111), likelihood = -79.736323.

## Discussion

EDP in the auks and penguins tend to be longer than predicted by egg mass (Figure 1). Long developmental periods are characteristic features of the life-histories of oceanic birds [5]. Here we have investigated the influence of several behavioural and ecological factors, proxies for nutrition and predation, on EDP in these taxa. Our results support the hypothesis that surrogates related to both nutrition and predation played important roles in shaping the evolution of EDP within alcids and penguins.

### Effect of behavioural and ecological variables on EDP

Firstly, the most important variable affecting EDP was clutch size. As predicted, larger clutches seem to drive the evolution of shorter EDP presumably due to an arms race between siblings for early hatching [6,18]. Among our taxa, most of the two-egg clutches were confined to the penguins; it is noteworthy that in auks the two-egg clutch is found exclusively among two clades: the guillemots, nearshore specialists that provision very frequently [38]; and the murrelets of the genus *Synthliboramphus *in which parents take their two chicks to sea, thus to the food source, within a day or two after hatching [28].

Secondly, EDP tends to be longer in those taxa that attend the colony nocturnally or crepuscularly rather than diurnally. Nocturnality is thought to have evolved in seabird groups primarily to reduce predation on adults while visiting colonies [28,39]. From the perspective of the offspring, nocturnal provisioning decreases the rate of postembryonic development because it restricts the number of energetically demanding [40] provisioning trips that each parent makes to one per day [27]. Given some direct or indirect mechanistic link between EDP and post-EDP, we can expect nocturnality to be associated with slower embryonic development. In the auks, the strong effect of nocturnality on EDP is perhaps most clearly evident in the Aethiini and Fraterculini: in both tribes, the basal species, including Cassin's auklet (*Ptychoramphus aleuticus*) and rhinoceros auklet (*Cerorhinca monocerata*), is nocturnal, delivers at most one meal per parent per day to the nest site [27,41], and has a very long EDP (and post-EDP).

Thirdly, we found an association between longer EDP and the use of enclosed, rather than open nest sites, an association also observed in other avian groups [42]. Like previous authors, we attribute this to the fact that offspring experience higher predation risk at open nest sites. There are few open-nesting auk species, but among these few, avian predators (i.e., larids and corvids) prey heavily on murre eggs and chicks at cliff sites [43,44], as do corvids on marbled murrelet eggs and chicks at nests on tree branches in old-growth forests [30]. Because most auks breed on islands free of terrestrial mammals [25], and because their fully enclosed nest sites are inaccessible to avian predators, egg predation is rare or non-existent under most natural conditions in burrow- and crevice-nesting auks [25]. On the other hand, relatively more penguins are open-nesting, and nest predation by skuas, gulls and petrels is a common occurrence [33].

Because of the mortality risk at open-topped nest sites, one of the two parents must always remain to guard the offspring throughout the period of post-EDP on the colony; or, analogously, adult penguins must guard the juveniles that assemble in crèches. That requirement for protection reduces the maximum provisioning rate at the nest [45]. Therefore, we might expect nesting habits to have opposing influences on EDP due to nutrition (longer) and predation risk (shorter). That murre EDP is brief relative to egg size for an auk (Figure 1) suggests that the mortality risk has been a very strong selective factor. It is interesting therefore that limits on the provisioning rate, rather than predation risk, usually are considered the primary drivers of the evolution of the unusually brief nestling periods in alcids [26,46,47], but see Cody [48].

Finally, our results further suggest that foraging habitats (nearshore or offshore) also help shape the evolution of auk EDP. This factor operated in the predicted direction: EDP tended to be briefer in taxa that feed in less distant, nearshore waters, which in seabirds facilitates increased rates of provisioning and post-embryonic growth [5,49]. However, the influence of this variable was not statistically significant in our results.

### Data quality and availability

In the present study, we have used behavioural and ecological surrogates for offspring growth and mortality (i.e., nutrition and predation), rather than direct measures such as are used widely in other studies; see for example Martin [14]. However, comprehensive vital rate estimates are available for relatively few species [25,32], and complete measures of growth rate are lacking entirely in auk clades in which offspring complete all (all four species of Synthliboramphini) or most (three of four species of Alcini) of their post-natal growth at sea. Moreover, the quality of the information is somewhat uneven (e.g., invasive techniques are required to study hole-nesting species, some of which are highly sensitive to disturbance [28,50]). Even within species for which a considerable amount of reliable information is available, such as the open-nesting murres (*Uria *spp.), which are widely studied throughout their range using standardized, non-invasive protocols [51], growth and mortality rates can vary dramatically from year to year and site to site [52]. The variation can be driven by a number of factors, including colony size and thus intraspecific competition [53] and oceanographic variability [54]. In sum, with the available data it simply is not feasible to use direct estimates, especially given that sample sizes for our analyses are already necessarily small.

### Other factors

One factor we would have liked to consider directly is adult survival rate. For example, we noted that species with high adult survival rates might be less attentive to their offspring, if attending the nest subjects them to an unacceptable mortality risk [7,55]. The key prediction of this hypothesis is that there is a positive correlation between the amount of egg neglect and the duration of EDP [19]. While this basic prediction is supported intra-specifically in the auks [56], the hypothesis is not tenable applied across species. Egg neglect is rare in the two auk clades with the longest EDPs for egg mass, the Aethiini and the Fraterculini [25]. Neglect is also rare (albeit slightly less so) in murrelets (*Brachyramphus *spp.) and guillemots (*Cepphus *spp.), and all but non-existent in murres (*Uria*. spp.), whose eggs would almost certainly be taken by predators or roll away from the nest site if left alone. In fact, egg neglect is common only in the murrelets (*Synthliboramphus *spp.), in which EDP is intermediate relative to egg mass. Thus, there is no simple positive association between frequency of egg neglect and EDP, and further, there also is no simple association between egg neglect and the suite of predictor variables used in our analysis.

More generally, the limited data available on adult survival rates are equivocal in relation to the idea that long lifespan is linked to slow development, thus long EDPs and their predictors. In the Aethiini, a reasonably well studied clade, adult survival averages 87% ± 4 (95% CI) in the diurnal least auklet (*Aethia pusilla*) [57] and 86% ± 2 in diurnal crested auklet (*Aethia cristatella*) [58]; but in nocturnal Cassin's auklet, with the longest EDP in the tribe, various studies document similar survival rates of 88% ± 5 [59]; 84% ± 4 in females and 0.75 ± 0.03 in males [60]; and 0.789 ± 0.040 (SE) and 0.774 ± 0.036 (SE) for males and females [61]. Likewise, within the Fraterculini, survival in the nocturnal rhinoceros auklet, with the longest EDP in the tribe, averaged 0.86 ± 0.02 (both sexes), lower in general than in the diurnal tufted puffin (*Fratercula cirrhata*), 0.96 ± 0.05 for females and 0.91 ± 0.06 for males [60]; and for diurnal, brief EDP Atlantic puffin (*Fratercula arctica*) survival was 89-99% in 19/20 years, but dropped to 81% in 1/20 years [62]. In aggregate, however, previous studies do as predicted indicate a particularly low adult survival rate relative to body mass in the guillemots (*Cepphus *spp.), in which parental investment appears to be particularly high due to their two-egg clutch, very high provisioning rates, and fast development [63].

The question remains as to what proximate mechanisms facilitate the evolutionary responses to selection on EDP. In the auks, eggshell porosity and EDP are negatively related, after controlling for egg size [64]. Thus, a reduction in the EDP might be achieved in part by providing the developing embryo with access to more oxygen to fuel its metabolism. Maternally derived yolk hormones also could be involved; there is some evidence that yolk testosterone levels are higher in avian species with briefer EDPs [65,66], but see Gill et al. [67]. However, to date there has been no investigation of the role of yolk hormones in the evolution of auk EDPs.

## Conclusion

Much current debate centers on the relative importance of nutrition and predation in avian life-history evolution [6,7,18]. Bennett and Owens [2] concluded that it was the adoption of different nest types, which largely determine predation risk, which acted as the primary catalyst for life-history diversification in ancient avian lineages. Food availability, they argued, was mainly involved in population regulation, rather than life-history diversification, a view that is in contrast with Lack [5]. We found evidence that both nutrition and predation have played roles in shaping the evolution of EDP at the lower taxonomic-level of the alcids and penguins. We suggest that a full understanding of the nature of avian life-history evolution will require additional studies that focus on lower-level taxonomic groups, especially those, like the taxa studied here, that exhibit marked interspecific variation in life-history traits. Further, speciose clades for which reliable data on key factors are available will generate conclusions based upon more robust sample sizes and lend stronger inference concerning the evolution of avian life-histories drawn from comparative studies. Future studies also could comprehensively investigate the roles of other ultimate factors, such as extra-pair fertilization rates [18] and parasites [8].

## Methods

### Phylogenetic inference

We collected nucleotide sequences for the mitochondrial cytochrome *b *locus from GenBank [68] and aligned these using BioPerl's [69] wrapper around MUSCLE [70], obtaining a multiple sequence alignment of 1144 columns. We then used reversible jump for selection of all prior parameters and the BayesPhylogenies program [71] to construct a Markov chain of trees, using a general time reversible [72] model of sequence evolution with 4 discrete gamma rate heterogeneity categories. We ran the chain for 3 * 10^6 ^generations, sampling topologies every 30,000^th ^generation and allowing a burn-in of 25% of the resulting chain, i.e. omitting the first 750,000 generations. We rooted the remaining trees on our putative outgroup, great crested grebe (*Podiceps cristatus*), which we then pruned from the trees. We subsequently built a Majority Rule consensus tree, which is the tree we used for the remainder of the analysis.

### Comparative analysis

We collected the following comparative data from the recent literature [25,32,73]: EDP in days, egg mass in grams, clutch size, foraging strategy (0 = inshore foraging, 1 = offshore foraging), activity pattern (0 = nocturnal or crepuscular, 1 = diurnal) and nest type (0 = enclosed nest, burrow, rock cracks or caves, 1 = open nests). For some of the taxa for which we found sequence data we were unable to locate comparative data: four penguin species including snares (*Eudyptes robustus*), royal (*Eudyptes schlegeli*), white-flippered (*Eudyptula albosignata*), and Galapagos (*Spheniscus mendiculus*) penguins, in addition to four alcids Craveri's (*Synthliboramphus craveri*), Kittlitz's (*Brachyramphus brevirostris*), long-billed (*Brachyramphus marmoratus perdix*) murrelet, and whiskered auklet (*Aethia pygmaea*). Based on the relationship of *expected EDP *= *17.18 × egg mass*^*0.119 *^for the Charadriiformes [74] we calculated the residuals of EDP from this slope (See Table 1).

Using our pruned consensus tree, we then analyzed the regression of EDP residuals on the combination of clutch size, foraging strategy, activity pattern and nest type using the continuous maximum likelihood regression method of the BayesTraits program [34]. The commonly-explored κ parameter (for stretching of long branches relative to short branches) and δ parameter (overall path length scaling) were omitted from the calculations reported here. We did estimate the λ parameter, which quantifies the influence of shared ancestry on the patterns of covariance among the taxa for a given trait. This parameter is used to test whether one of the underlying assumptions in comparative analysis holds: that species values are not independent for a given tree and trait. Values of λ near 1.0 are interpreted to mean that the Brownian motion model is a correct representation of the data.

The comparative analysis reports the standardized coefficients (sometimes called β) and the standard error for each of the independent variables. The biological interpretation of the effect of each independent variable on EDP follows from the sign of the coefficient, and the t-test statistic is calculated from its division by the standard error (degrees of freedom are the number of taxa) (C. Venditti, pers. comm.).

## Authors' contributions

JMH conceptualized the study; JMH and JBJ collected life-history data; KBG and RAV collected cytochrome *b *sequence data; RAV developed phylogenetic relationships and performed comparative analysis. All authors contributed to the writing and approved the final manuscript.
